# Non-Invasive Blood Pressure Sensing via Machine Learning

**DOI:** 10.3390/s23198342

**Published:** 2023-10-09

**Authors:** Filippo Attivissimo, Vito Ivano D’Alessandro, Luisa De Palma, Anna Maria Lucia Lanzolla, Attilio Di Nisio

**Affiliations:** Department of Electrical and Information Engineering, Polytechnic University of Bari, 70125 Bari, Italy; filippo.attivissimo@poliba.it (F.A.); v.dalessandro4@phd.poliba.it (V.I.D.); luisa.depalma@poliba.it (L.D.P.); attilio.dinisio@poliba.it (A.D.N.)

**Keywords:** blood pressure (BP), digital health, machine learning (ML), physiological monitoring

## Abstract

In this paper, a machine learning (ML) approach to estimate blood pressure (BP) using photoplethysmography (PPG) is presented. The final aim of this paper was to develop ML methods for estimating blood pressure (BP) in a non-invasive way that is suitable in a telemedicine health-care monitoring context. The training of regression models useful for estimating systolic blood pressure (SBP) and diastolic blood pressure (DBP) was conducted using new extracted features from PPG signals processed using the Maximal Overlap Discrete Wavelet Transform (MODWT). As a matter of fact, the interest was on the use of the most significant features obtained by the Minimum Redundancy Maximum Relevance (MRMR) selection algorithm to train eXtreme Gradient Boost (XGBoost) and Neural Network (NN) models. This aim was satisfactorily achieved by also comparing it with works in the literature; in fact, it was found that XGBoost models are more accurate than NN models in both systolic and diastolic blood pressure measurements, obtaining a Root Mean Square Error (RMSE) for SBP and DBP, respectively, of 5.67 mmHg and 3.95 mmHg. For SBP measurement, this result is an improvement compared to that reported in the literature. Furthermore, the trained XGBoost regression model fulfills the requirements of the Association for the Advancement of Medical Instrumentation (AAMI) as well as grade A of the British Hypertension Society (BHS) standard.

## 1. Introduction

Hypertension is a health condition in which blood pressure (BP) at rest is higher than the physiological standards for a long time. It is one of the most common diseases; in fact, it affects about 20% of the adult population, representing one of the major clinical problems, and it is associated with chronic diseases and an increase in mortality and morbidity. BP is related to the force that blood exerts against the walls of blood vessels due to the pumping action carried out by the heart and its value depends on various factors. Moreover, BP is one of the so-called vital signs, also including respiratory rate, heart rate (HR), oxygen saturation (SpO_2_), and body temperature, which require adequate monitoring on the general population.

For this reason, there is the spread of the development of practical and reliable telemedicine solutions [1,2,3,4] to guarantee monitoring at home and at hospital with the aim of ensuring early identification and prevention of cardiovascular diseases, hypertension, and other related diseases. As concerns BP measurement, traditional cuff-based devices have several disadvantages because they are not always accurate, they need appropriate calibration, and they do not allow continuous monitoring since performing a measurement requires about one minute or more. On the contrary, there is a strong tendency today to monitor health at home by using wearable, affordable, and small devices that are simple to use, non-invasive, and even wireless to obtain measurements continuously [5,6,7]. Hence, researchers are investigating ways to perform cuff-less and non-invasive BP measurements. As a matter of fact, the monitoring of the health of individuals is also made possible by the spread of artificial intelligence in healthcare [8,9,10].

Nowadays, a measurement technique that is spreading for real-time monitoring of vital signs is photoplethysmography (PPG) [11,12,13,14]. Indeed, PPG is a simple, low-cost, and non-invasive optical measurement method that, in addition to the estimation of HR, SpO_2_, and respiration rate, provides important health information regarding atherosclerosis and arterial stiffness. It is a type of plethysmography (PG) that exploits optical properties unlike other types of PG, such as those based on capacitive, inductive, and piezoelectric properties [15,16].

Recently, the use of PPG to also estimate BP values has become an active area of research. However, quite often, studies have focused on the simultaneous use of both electrocardiogram (ECG) and PPG signals or on the use of multi-site PPG acquisition [17,18] which introduces system complexity and the need for synchronization between those signals [19]. In fact, PPG for the estimation of BP presents criticalities and limitations, such as the development of multi-photodetectors, noise elimination, the event detection, the need of individual calibration, and calibration drift. A useful algorithm that can be used to overcome motion artifacts’ problems is the adaptive neuro fuzzy inference system (ANFIS) that allows improvements in the signal to be obtained [20]. Moreover, this algorithm has proved to be versatile for other fields of application [21].

As a matter of fact, the single-site PPG signal approach has great potential even though it has some criticalities and limitations. Its deployment has also increased thanks to the encouraging results obtained by exploiting machine learning (ML) algorithms trained on purposely selected PPG signal features [22,23,24,25,26,27,28].

In a previous work carried out by the authors [29], PPG signals were analyzed to select the most significant features for BP estimation by using several selection algorithms, i.e., RReliefF [30,31], Correlation-based Feature Selection (CFS), and Minimum Redundancy Maximum Relevance (MRMR) [32,33]. That methodology has led to the justification of the application of the Maximal Overlap Discrete Wavelet Transform (MODWT) to enhance the single-site PPG signal and to the selection of new proposed features [29]. Following this line of research, in this paper, our focus is on the actual development of ML techniques to find the best algorithm to measure BP, showing the usefulness of the already analyzed features and, in particular, those selected by means of MRMR, including those obtained after the enhancement with MODWT. The novelty of the research is in the use of new extracted features from PPG signals, whose significance was evaluated by using several criteria, and in the use of ML algorithms. 

For this purpose, eXtreme Gradient Boost (XGBoost) models with Bayesian optimization and Neural Network (NN) models were trained for regression using significant features selected with the MRMR algorithm. A comparison of results between XGBoost and NN models was presented and the improvements with respect to the literature, by using XGBoost models and the proposed features, are shown.

The paper is structured as follows: in Section 2, the description of the dataset used to train ML models is provided; in Section 3, the ML approach for both XGBoost and NN models is presented; in Section 4, the results obtained using the best model are reported and compared with the literature, focusing on standard medical protocols for performance assessment; and finally, there are the conclusions.

## 2. Dataset

**Figure 1:**
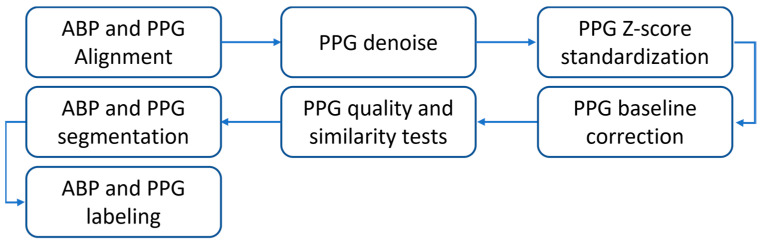
Workflow of the processing steps.

In this work, the MIMIC-III Waveform Database [34,35,36] was used to obtain the dataset for training and validation following the same processing reported in detail in [29]. The MIMIC-III Waveform Database is a large and open access database where protected health information has been deidentified. It includes waveform records of digitized signals acquired at 125 Hz, such as arterial blood pressure (ABP) measured invasively, PPG, ECG, and respiration for neonatal and adult patients admitted to intensive care units and monitored with iMDsoft MetaVision ICU or Intellivue MP-70 monitors. Among these acquired data, ABP and PPG signals have proved useful for our work. Many processing steps, shown in Figure 1, were performed such as alignment between ABP and PPG signals, pre-processing of PPG signals with denoising, Z-score standardization, baseline correction, quality, similarity tests, and ABP and PPG pulses segmentation and labeling.

After the processing briefly described above, the features presented in [29] were calculated on the PPG signal. Many features were extracted in the time and frequency domain, others were related to the amplitude of the characteristic points (max slope point, systolic peak, dicrotic notch, inflection point, and diastolic peak), times and durations of characteristic points, areas, non-linear functions (logarithm of positions of dicrotic notch and inflection point), statistics (mean, STD, skewness, percentiles) and first and second derivatives. In this way, a dataset has been created containing, for each PPG pulse, 195 features and the target values of systolic blood pressure (SBP) and diastolic blood pressure (DBP) measured on the ABP signal. Then, the dataset was reduced to SBP in the range 80 mmHg to 180 mmHg and DBP in the range 60 mmHg to 110 mmHg to facilitate comparisons of the literature because similar distributions are used in other works [22,23,26,27,37,38,39,40]. Indeed, SBP under 80 mmHg and DBP under 60 mmHg correspond to a severe hypotension condition while SBP over 180 mmHg and DBP over 110 mmHg correspond to a severe hypertension condition and, in these cases, there were few observations in the initial dataset. 

**Figure 2:**
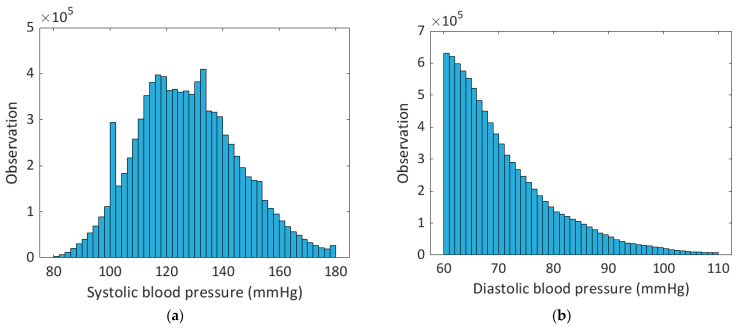
(**a**) Systolic and (**b**) diastolic blood pressure occurrences in 2 mmHg bins. Only the observations with 80 mmHg ≤ SBP ≤ 180 mmHg and 60 mmHg ≤ DBP ≤ 110 mmHg were considered since outside these ranges there were few observations and, also, DBP less than 60 mmHg corresponds to a severe hypertension condition.

At the end of the processing, performed in MATLAB R2022a, the dataset contained 9.1 × 10^6^ observations of PPG pulses from 1080 patients. The distribution of systolic and diastolic blood pressure values of the dataset processed in this work are shown in Figure 2. The described dataset was used to train and validate ML models developed in Python language, as discussed in the following sections. The dataset used to train and validate ML models included 9 × 10^6^ observations; of these, the 90% constituted the training set and the 10% constituted the validation set. Instead, the test set included 100,000 observations.

## 3. Machine Learning Models

ML offers powerful techniques to identify and evaluate cardiovascular risk and health conditions. In this paper, it has been exploited to train supervised regression models able to measure BP starting from features extracted from the PPG signal. For training purposes, each observation of the dataset is provided with systolic and diastolic labels obtained from the corresponding ABP signal, which serves as ground truth, as reported in [29]. 

In this paper, an XGBoost model was trained because of advantages such as execution speed and model performance, which have turned out to be suitable for our goal, while an NN model was trained to carry out a comparison of the results and it was chosen because it is an approach common to several researchers [27,28,37,41] and is characterized by higher training speed. Moreover, XGBoost models were used in the literature for a variety of purposes, such as wearable running monitoring [42], but recently also for PPG signal processing to estimate blood glucose levels [43], blood pressure (by using multisite PPG acquisition and Pulse Transit Time features) [44], and vascular aging [45].

XGBoost is an efficient open-source implementation of the gradient boosting algorithm and is also available in Python using the Scikit-learn library utilized in this work. Overall, gradient boosting refers to a class of ensemble ML algorithms that can be used both for classification and regression; ensembles, as a matter of fact, are based on decision tree models. In fact, trees are added to the ensemble to correct prediction errors made previously and these models are fitted using a differentiable loss function and a gradient descent optimization algorithm in order to minimize the loss gradient; moreover, this algorithm provides hyperparameters that can be tuned, such as the number of trees or estimators, the learning rate, the row and column sampling rate, the maximum tree depth, the minimum tree weight, and the regularization terms alpha and lambda. Indeed, XGBoost adds a regularization term in the objective function to make the model less vulnerable to overfitting. 

Moreover, in this work, Bayesian hyper-parameter optimization [46] was used to tune the hyper-parameters of the XGBoost model in the chosen search space. Bayesian optimization allows the optimization of a proxy function rather than the true objective function and the search balances the exploration against exploitation, so at the beginning, it randomly explores to build the surrogate function with the objective of minimizing the cost function at a global level. In this work, the Bayesian Optimization implementation offered by the Python library Scikit-optimize was used. The Root Mean Square Error (RMSE) evaluation metric was defined using a Scikit-learn function to allow the conversion of optimization into a minimization problem as required by Scikit-optimize.

The Bayesian optimization was set providing the basic regressor, the search space, the evaluation metric, the cross-validation strategy (chosen to be 7-fold), the max number of trials, and the optimizer parameters for which the Gaussian Process (GP) was used. Then, the best hyper-parameters were obtained and used to instantiate the XGBoost model to be trained using the 10-fold cross-validation. 

In the next paragraphs, there will be a focus on the XGBoost and NN models that were trained.

### 3.1. XGBoost Models

For both SBP and DBP, the entire dataset was used. The training and cross-validation were made using 9 × 10^6^ observations (out of 9.1 × 10^6^ observations). In total, 20 features for SBP and 25 features for DBP were used and selected in order of highest MRMR score among the 195 features listed in [29], which include those derived from the MODWT enhanced PPG signal. The number of features used to train the models has been chosen using the RReliefF algorithm for systolic and diastolic cases. In fact, using the RReliefF algorithm, the 20 features for SBP and the 25 features for DBP have an importance score greater than 0.001. We have considered lower scores as not significant because lower values are related to uncorrelated features to the output. That reduction in the number of features was operated to decrease the complexity of models and training; as a matter of fact, removing the noisy features helps with memory and computational cost but also helps avoid overfitting. Moreover, a normalization of columns into the range [0, 1] was carried out before the training.

Then, the first step consisted of finding of the best hyper-parameters in a specified search space for the Bayesian optimization using the selected features for both SBP and DBP measurements.

The search spaces and the best hyper-parameter values for SBP and DBP measurements are, respectively, shown in Table 1 and Table 2.

An explanation of XGBoost hyper-parameters is reported below. The learning rate is the step size shrinkage used for the update to make the model more robust and to prevent overfitting by shrinking the feature weights; it is chosen in the range [0, 1] with typical values in [0.01, 0.2]. The maximum depth of a tree is used to control over-fitting as higher depth will make the model more complex and more likely to overfit; the value 0 is only accepted in a loss-guided growing policy while large values bring an aggressive consumption of memory. Any positive value is admissible, with typical values in [3, 10]; in this work, trial and error was used to modify the upper bound of the range to obtain better results. The subsample is, instead, the fraction of observations to be randomly sampled for each tree and is useful to prevent overfitting; in fact, lower values make the algorithm more conservative while too small values might lead to under-fitting. For this reason, the range is [0, 1] and typical values are in [0.5, 1]. The subsample ratio of columns by tree is the subsample ratio of columns when constructing each tree; this parameter has a range of [0, 1] and the default value of 1. Lambda is the L2 regularization term on weights and the increase in this value makes the model more conservative while Alpha is the L1 regularization term on weights and it is used in case of very high dimensionality so that the algorithm runs faster when implemented. Finally, estimators are the number of trees in an XGBoost model.

For the three last hyper-parameters, a trial and error method was used to define the range.

### 3.2. NN Models

**Figure 3:**
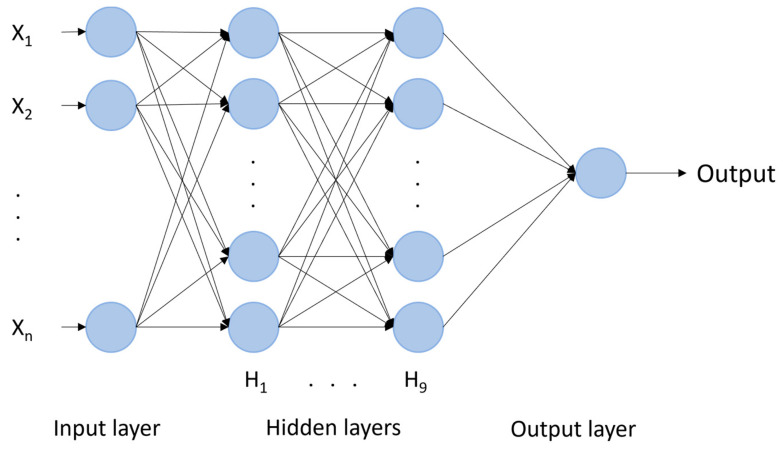
NN with nine hidden layers with 1024, 1024, 1024, 512, 512, 512, 128, 64, and 64 neurons.

In Python, TensorFlow 2.9.1 was used to define a sequential model with an input layer of size n, nine hidden layers, and an output layer. For all the layers, the activation function chosen was the Rectified Linear Unit (ReLU). The number of hidden layers and of neurons has been set making several trials. The NN model is shown in Figure 3. For SBP estimation, n = 20 while for DBP estimation, n = 25.

Moreover, several optimizers were tested such as Adadelta, Adagrad, Adam, Adamax, Nadam, RMSprop, and SGD but the best result for both SBP and DBP estimations was obtained using the Nesterov-accelerated Adaptive Moment Estimation (Nadam) algorithm. 

The fit was made using a batch size of 4096, 150 epochs, and a validation split of 0.2.

The NN architecture was chosen after trials and errors, by adding hidden layers since there was not an improvement in the results. The ReLU activation function was chosen because it is suitable for the normalized inputs and this function has allowed better results to be obtained. The batch size needs to fit the memory requirements of the GPU and the architecture of the CPU since too low values did not perform well while too high values were not allowed considering the memory requirements. Hence, the maximum possible batch size was set. The number of epochs was chosen in the range [50, 200] but beyond the 150 epochs there were not improvements.

## 4. Results and Discussion

In this section, the performance of ML algorithms will be shown. The two models, XGBoost and NN, were trained using the features selected by the MRMR algorithm and these features also include the new ones obtained on MODWT enhanced PPG pulses as reported in [29]. As pointed out in that previous work, it has been found that by using the MODWT, the PPG signal is enhanced, with an improvement in the identification of the characteristic points and making it more similar to the ABP signal.

The criteria used to evaluate the performance of ML models for estimating BP are the RMSE, Mean Absolute Error (MAE), correlation coefficient (R), and Mean Error (ME). 

The results were then compared with other methods reported in the literature as well as with BP measurements standard guidelines focused on the classification of hypertension states. The predicted BP values from the regression model and the true values were used to verify the correct classification into the seven classes defined by the guideline considering the range of values of SBP and DBP. The classification results are evaluated by means of a confusion matrix.

### 4.1. Training and Test of XGBoost and NN Models

In this paragraph, the results obtained after training and validation are reported. In Table 3, XGBoost and NN results are reported considering the RMSE and MAE.

After validation, a test was made for both models using a set of 100,000 new observations (out of the entire dataset of 9.1 × 10^6^ observations) not included in the training set. The results were reported in Table 4 in which performance parameters are reported for SBP, DBP, and Mean Arterial BP (MAP).

In addition to SBP and DBP, MAP was considered because it is linked to the total peripheral resistance and to cardiac output and is associated with HR [47,48]. MAP is a popular BP parameter, and it is defined as the average pressure of the artery of a subject during one cardiac cycle (1). It is considered as a better indicator of perfusion to vital organs when compared with SBP [49].

(1) $$MAP=\frac{SBP+2\cdot DBP}{3},$$

The results reported in Table 3 and Table 4 show that the use of XGBoost models rather than NN allows better results for both systolic and diastolic pressure measurement to be obtained. 

Moreover, the results for XGBoost models obtained in the final test phase, shown in Table 4, are similar and confirm the ones obtained during the training and cross-validation phase, shown in Table 3.

**Figure 4:**
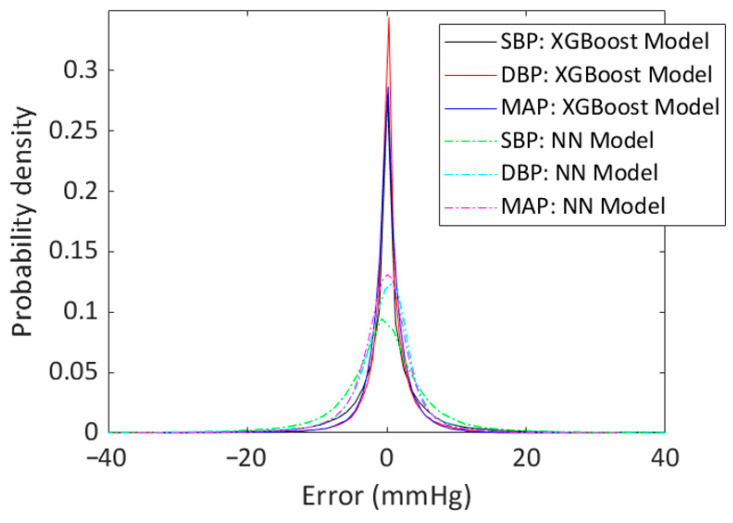
Error probability density of SBP, DBP, and MAP estimations. Errors were defined as the difference between the predicted pressures (using XGBoost model or NN model) and measured ones; then, their histograms were normalized to obtain the probability densities shown in the plot.

**Figure 5:**
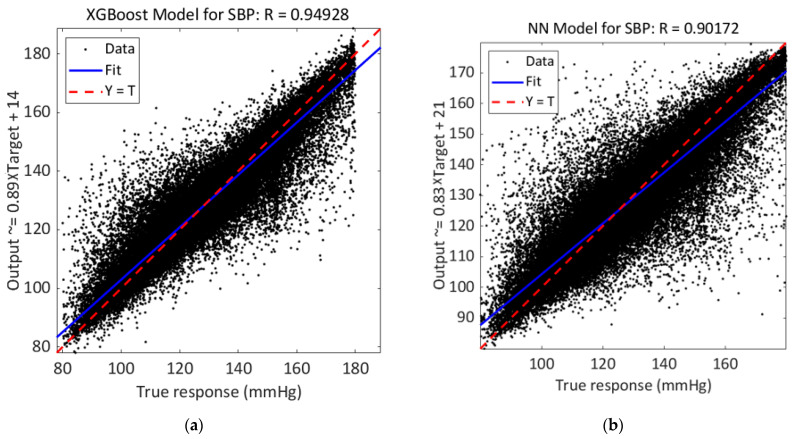

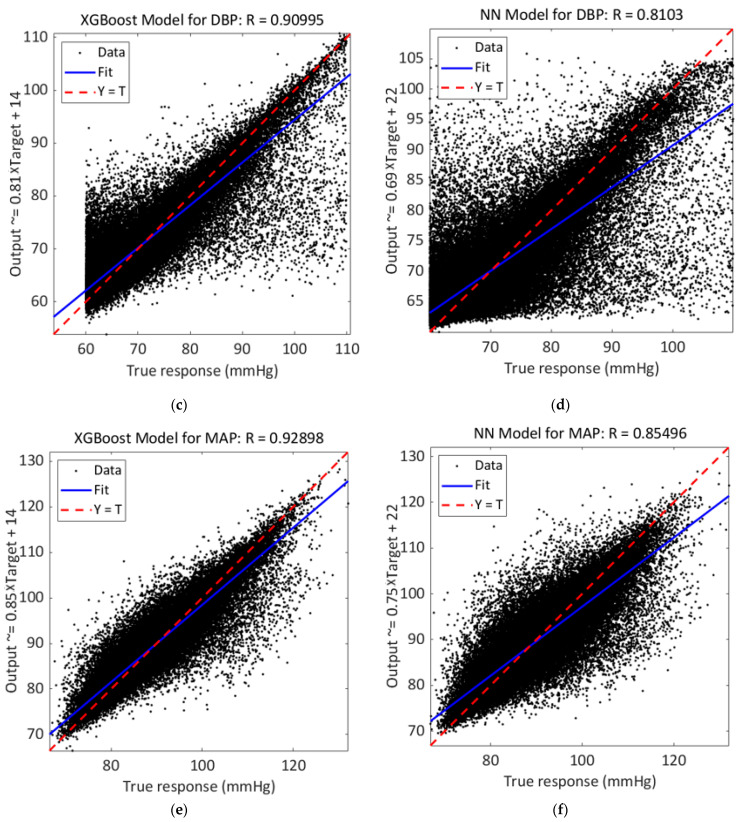
(**a**,**c**,**e**) Regression of the predicted output and true response for SBP, DBP, and MAP estimations using the XGBoost model; (**b**,**d**,**f**) Regression of the predicted output and true response for SBP, DBP, and MAP estimations using the NN model.

Error probability densities of SBP, DBP, and MAP estimations are shown in Figure 4, where it is possible to notice that errors obtained using the XGBoost model have a narrower and more concentrated distribution around zero than the distribution obtained using the NN model. From regression plots reported in Figure 5, it is possible to notice that best predictions are obtained by using XGBoost models; in fact, R is in the three cases higher than those obtained using NN models.

During the training phase, it was noticed that the training time for NN was smaller than the training time for the XGBoost models. The inference time was significantly reduced for XGBoost models so considering this aspect, it is possible to use the trained model for real time predictions useful for continuous monitoring.

Considering the computational complexity of current implementations for features extraction and ML models, onboard processing on a wearable device is not viable. So, a cloud-based solution would be required. The future aim is to streamline feature extraction by including only those selected in the present study and simplify models to permit onboard processing, reducing the computational complexity and assessing the minimal hardware requirements.

### 4.2. Comparison with Other Methods

A comparison of results with the literature is difficult due to the different evaluation criteria and the different datasets. In this paper, the type of the algorithms and the use of features have been used as the criteria to select and identify other works in the literature to make a comparison. In this context, the criterion is the training of ML algorithms with features extracted from the PPG signal, namely, the research’s methodology.

In Table 5, the performance of other methods is shown.

The comparison with other works has shown that our models, based on the use of XGBoost, the MRMR selection algorithm, and features obtained on MODWT, enhanced PPG pulses, obtained small estimation errors for both systolic and diastolic blood pressure measurements [29]. In fact, XGBoost is derivative-free so it might have some advantage when the fitting problem has a lot of degrees of freedom. Moreover, the use of MODWT enhancement has allowed characteristic points of PPG pulses such as the diastolic point to be emphasized; these two aspects can be decisive in obtaining such results. As a matter of fact, for SBP measurement, the proposed method has allowed a smaller RMSE compared to the other works reported in the Table 5 to be obtained. Obviously, as mentioned at the beginning of this section, a comparison of results is difficult; in fact, as reported in Table 5, different datasets were used as well as different ML algorithms. For example, it should be noted that Chowdhury et al. [24] obtained a smaller RMSE for DBP, which may depend on the different dataset used and on the use of demographic features that are a powerful means to predict BP values because gender, age, and height are related to the shape of the PPG pulses and to the arterial stiffness. Considering Zhang et al. [57], they use a GBR algorithm obtaining slightly worse results than those reported in this paper as well as in Fleischhauer et al. [55] using XGBoost; as a matter of fact, in this paper, the best results are obtained implementing the Bayesian optimization for our XGBoost models and a different selection of features also obtained after the MODWT enhancement. This seems to be a better solution also compared with other ML algorithms as reported in Table 5.

### 4.3. Compliance to Standards and Classification Guidelines

The correct estimation of BP is critical for the detection of states of hypertension and health status and hence, accuracy requirements for BP measurement devices and methods have been standardized. 

In this paper, the protocols proposed by the Association for the Advancement of Medical Instrumentation (AAMI) [58,59] and by the British Hypertension Society (BHS) [60] were considered to make a comparison with results reported in this paper as also made in [23,24,25,26,61,62,63]. 

Since the best results in this paper were obtained using the XGBoost models rather than using the NN models, the following comparisons regard only the XGBoost models. 

As shown in Table 6, Table 7, Table 8 and Table 9, the proposed method is compliant to AAMI and BHS grade A standards. The dataset included 1080 patients and a total of 9.1 × 10^6^ observations of PPG pulses.

As is possible to notice in Table 6 and Table 7, our results fulfill AAMI standard requirements; indeed, according to this protocol, the mean and the STD of the errors for both SBP and DBP estimations should not be more than 5 mmHg and 8 mmHg, respectively. Requirements of the BHS standard are also satisfied since the absolute error of more than 60% of the data is less than 5 mmHg, hence the method is considered as Grade A.

Moreover, as established in [26], another guideline was used to evaluate our regression models; for this purpose, the guideline [64] provided by the European Society of Hypertension (ESH) and the European Society of Cardiology (ESC) was considered. This guideline is focused on the state of hypertension and, in fact, categorizes it into seven classes:

- Optimal: if SBP < 120 mmHg and DBP < 80 mmHg;
- Normal: if 120 mmHg ≤ SBP ≤ 129 mmHg and/or 80 mmHg ≤ DBP ≤ 84 mmHg;
- High Normal: if 130 mmHg ≤ SBP ≤ 139 mmHg and/or 85 mmHg ≤ DBP ≤ 89 mmHg;
- Grade 1 Hypertension: if 140 mmHg < SBP ≤ 159 mmHg and/or 90 mmHg ≤ DBP ≤ 99 mmHg;
- Grade 2 Hypertension: if 160 mmHg ≤ SBP ≤ 179 mmHg and/or 100 mmHg ≤ DBP ≤ 109 mmHg;
- Grade 3 Hypertension: if SBP ≥ 180 mmHg and/or DBP ≥ 110 mmHg;
- Isolated Systolic Hypertension: if SBP ≥ 140 mmHg and DBP < 90 mmHg.

**Figure 6:**
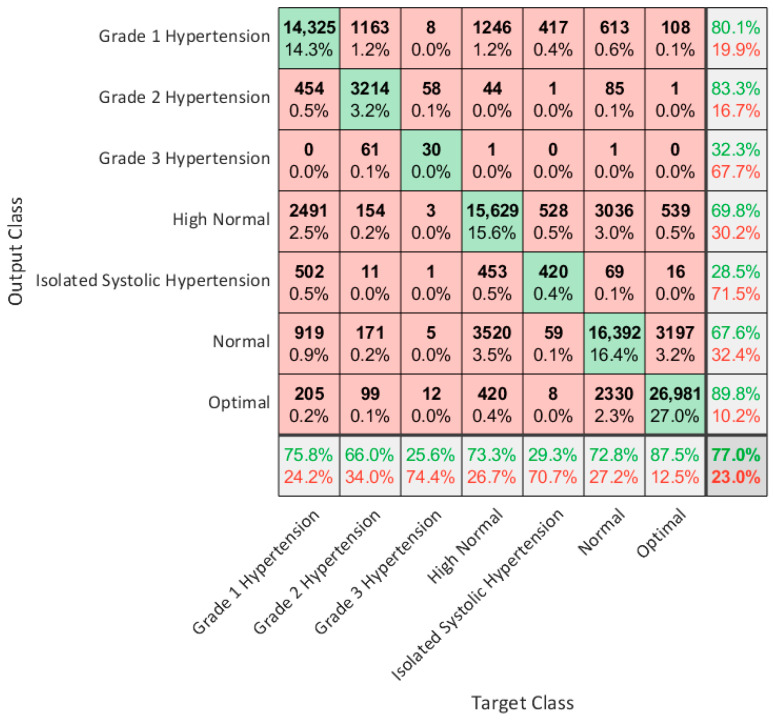
Confusion matrix for BP level classification according to ESH/ESC guidelines.

Since hypertension is a state of health of interest to be identified, we also used ESH/ESC guidelines to evaluate our regression models with a classification of the predicted values into seven classes. The BP ground truth and the BP predicted by the XGBoost model were labeled according to the previously described classification to evaluate the consistency between the classified predicted values and the classified true values in the different states of hypertension. The results are shown in Figure 6 and in Table 10. In the table, the accuracy, sensitivity, specificity, and F1-score are provided. There are two classes with a low sensitivity that are “Grade 3 Hypertension” and “Isolated Systolic Hypertension”. The low sensitivity is due to the few training cases in the dataset. Indeed, “Grade 3 Hypertension” is a critical condition while “Isolated Systolic Hypertension” has low frequency in young and middle-aged subjects.

As is possible to see in Table 10, the average of accuracy, sensitivity, specificity, and F1-score are, respectively, 90.3%, 76.9%, 93.5%, and 77.0%.

### 4.4. Bland–Altman Analysis

**Figure 7:**
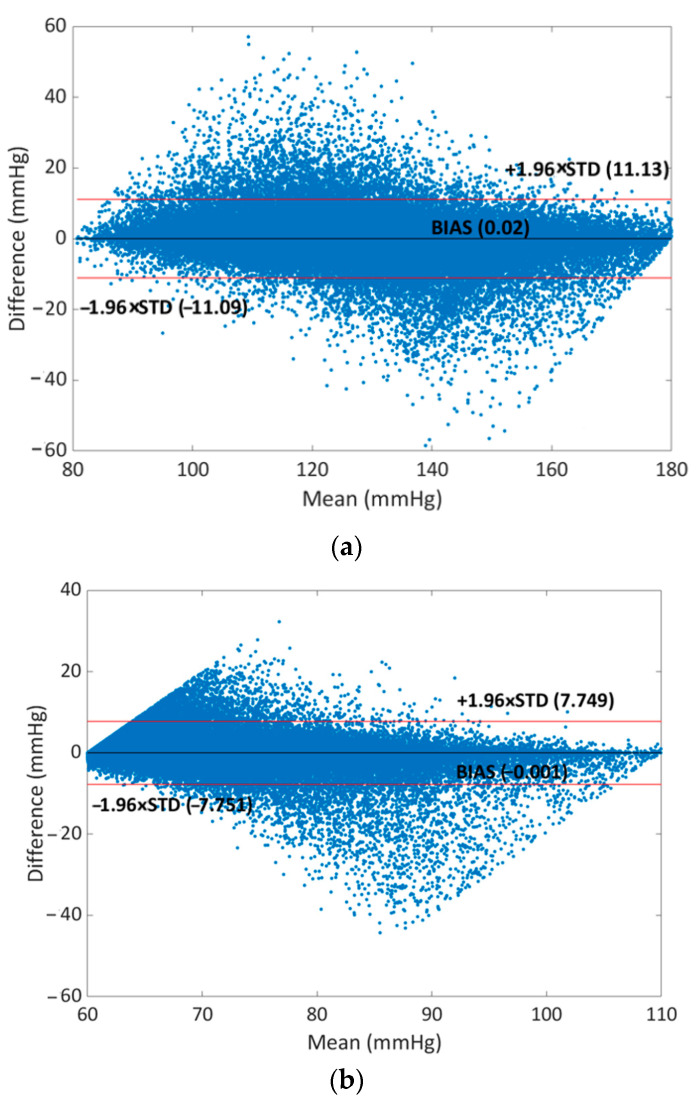

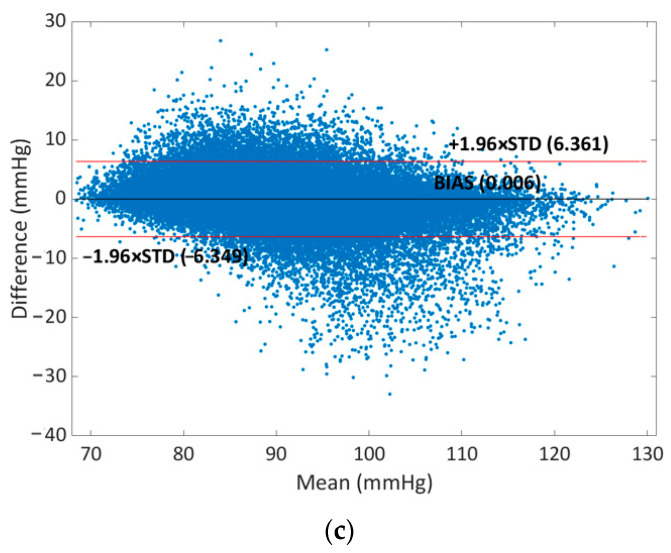
Bland–Altman plots for (**a**) SBP, (**b**) DBP, and (**c**) MAP.

Finally, to test the validity of the prediction of the XGBoost models for SBP, DBP, and MAP, a Bland–Altman analysis was performed which was used to determine the limits of agreement (LOA) between two different measurements in clinical practice [65,66]. The mean and STD of the differences between two measurements are used for statistical limits. The mean bias (mean of the differences) and its LOA are provided by the Bland–Altman plot that is shown in Figure 7. 

The black line represents the mean of differences (BIAS) while the red lines represent the upper and lower limits (BIAS ± 1.96 × STD) of the LOA [67]. The LOA for errors of SBP is [−11.09, 11.13] mmHg and the percentage of points outside the LOA is 5.91%; the LOA for errors of DBP is [−7.75, 7.75] mmHg with a percentage of 5.08% points outside while for MAP the LOA for errors is [6.35, 6.36] mmHg with a percentage of 5.73% points outside. So, considering these results, it is possible to confirm the good accuracy of the proposed model.

## 5. Conclusions

The possibility of measuring BP by using PPG signals is advantageous for the monitoring of this vital sign since it avoids the use of cumbersome cuff-based devices, and it allows continuous monitoring. However, PPG for the estimation of BP has several criticalities and limitations, such as noise elimination, individual calibration, and calibration drift, that must be overcome.

In our previous work [29], the focus was on the extraction of new features from PPG signals, including those obtained after the enhancement with MODWT, whose significance was evaluated by using several criteria, such as MRMR. In this paper, the features selected by the MRMR algorithm were used to train ML models to estimate BP, giving improved results.

Among the ML models, the XGBoost model with Bayesian optimization proved to be suitable for estimating purposes, giving better results than an NN model trained on the same data; as a matter of fact, the XGBoost model combined with the use of novel features allowed an improvement for systolic blood pressure measurement with respect to the literature.

In addition, the SBP and DBP estimators proved to fulfill the requirements of the AAMI and BHS grade A standards, but also, good classification results were obtained according to the ESH/ESC guideline. 

Considering these results, future work will focus on the realization of a portable measurement device to acquire PPG signals and implement the proposed BP estimator permitting onboard processing by reducing the computational complexity and assessing the minimal hardware requirements. 

## Figures and Tables

**Table 1:** Search spaces and best values of hyper-parameters for SBP.

Hyper-Parameter	Range	Best
Learning rate	[0.01, 1.0]	0.226
Maximum tree depth	[2, 15]	15
Subsample	[0.1, 1.0]	0.894
Subsample ratio of columns by tree	[0.1, 1.0]	1.0
Lambda	[1 × 10^−10^, 200]	120.0
Alpha	[1 × 10^−10^, 200]	1 × 10^−10^
Estimators	[50, 5100]	5000

**Table 2:** Search spaces and best values of hyper-parameters for DBP.

Hyper-Parameter	Range	Best
Learning rate	[0.01, 1.0]	0.136
Maximum tree depth	[2, 20]	15
Subsample	[0.1, 1.0]	0.894
Subsample ratio of columns by tree	[0.1, 1.0]	1.0
Lambda	[1 × 10^−9^, 200]	120.0
Alpha	[1 × 10^−10^, 200]	1 × 10^−10^
Estimators	[50, 6000]	5200

**Table 3:** Validation results for SBP and DBP estimations.

Model		RMSE (mmHg)	MAE (mmHg)
XGBoost	SBP	5.60	3.11
	DBP	3.92	2.09
NN	SBP	7.80	5.00
	DBP	5.56	3.53

**Table 4:** Test results using XGBoost and NN models.

Model		RMSE (mmHg)	MAE (mmHg)	R	ME (mmHg)
XGBoost	SBP	5.67	3.12	0.95	0.020
	DBP	3.95	2.11	0.91	−0.001
	MAP	3.24	2.01	0.93	0.006
NN	SBP	7.81	5.00	0.90	−0.420
	DBP	5.60	3.55	0.81	−0.250
	MAP	4.56	3.12	0.85	−0.310

**Table 5:** Comparison with other works.

Work	Method	Data Size	Performance Evaluation	SBP	DBP
Kachuee et al. [37]	Support vector machine (SVM)	MIMIC II (1000 subjects)	RMSE	/	/
			MAE	12.38	6.34
			R	/	/
			ME	/	/
Kim et al. [50]	ANN	180 recordings, 45 subjects	RMSE	/	/
			MAE	4.53	/
			R	/	/
			ME	/	/
Cattivelli et al. [51]	Proprietary algorithm	MIMIC database (34 recordings, 25 subjects)	RMSE	8.37	5.92
			MAE	/	/
			R	/	/
			ME	/	/
Zhang et al. [52]	SVM	7000 samples from 32 patients	RMSE	/	/
			MAE	11.64	7.62
			R	/	/
			ME	/	/
Zadi et al. [53]	Autoregressive moving average (ARMA) models	15 subjects	RMSE	6.49	4.33
			MAE	/	/
			R	/	/
			ME	/	/
Chowdhury et al. [24]	Gaussian process regression (GPR)	222 recordings, 126 subjects	RMSE	6.74	3.59
			MAE	3.02	1.74
			R	0.95	0.96
			ME	/	/
Hasanzadeh et al. [26]	AdaBoost	MIMIC II 942 subjects	RMSE	/	/
			MAE	8.22	4.17
			R	0.78	0.72
			ME	0.09	0.23
Kachuee et al. [38]	AdaBoost	1000 subjects	RMSE	/	/
			MAE	8.21	4.31
			R	/	/
			ME	/	/
Wang et al. [54]	ANN	58,795 PPG samples	RMSE	/	/
			MAE	4.02	2.27
			R	/	/
			ME	/	/
Kurylyak et al. [28]	ANN	15,000 PPG heartbeats	RMSE	/	/
			MAE	3.80	2.21
			R	/	/
			ME	/	/
Fleischhauer et al. [55]	XGBoost	MIMIC, Queensland, PPG BP (273 subjects and 259,986 single beats)	RMSE	/	/
			MAE	6.366	/
			R	0.874	/
			ME	/	/
Liu et al. [56]	SVR	MIMIC II 910 good PPG pules cycles	RMSE	/	/
			MAE	8.54	4.34
			R	/	/
			ME	/	/
Zhang et al. [57]	Gradient Boosting Regressor (GBR)	MIMIC II 2842 samples from 12,000 data points	RMSE	/	/
			MAE	4.33	2.54
			R	/	/
			ME	/	/
**Proposed method**	**XGBoost**	**MIMIC III** **9.1 × 10^6^ PPG pulses** **from 1080 subjects**	**RMSE**	**5.67**	**3.95**
			**MAE**	**3.12**	**2.11**
			**R**	**0.95**	**0.91**
			**ME**	**0.01**	**0.02**

**Table 6:** Comparison of results for the validation set with AAMI standard.

		ME (mmHg)	STD (mmHg)
Results	SBP	0.009	5.60
	DBP	0.019	3.92
	MAP	0.0157	3.21
AAMI	SBP	≤5	≤8
	DBP		

**Table 7:** Comparison of results for the test set with AAMI standard.

		ME (mmHg)	STD (mmHg)
Results	SBP	0.020	5.67
	DBP	−0.001	3.95
	MAP	0.006	3.24
AAMI	SBP	≤5	≤8
	DBP		

**Table 8:** Comparison of results for the validation set with BHS standard.

		Cumulative Error Percentage		
		≤5 mmHg	≤10 mmHg	≤15 mmHg
Results	SBP	80.85%	93.00%	96.84%
	DBP	89.56%	96.86%	98.74%
	MAP	90.89%	98.18%	99.49%
BHS	Grade A	60%	85%	95%
	Grade B	50%	75%	90%
	Grade C	40%	65%	85%

**Table 9:** Comparison of results for the test set with BHS standard.

		Cumulative Error Percentage		
		≤5 mmHg	≤10 mmHg	≤15 mmHg
Results	SBP	80.96%	92.91%	96.73%
	DBP	89.48%	96.87%	98.68%
	MAP	90.84%	98.07%	99.44%
BHS	Grade A	60%	85%	95%
	Grade B	50%	75%	90%
	Grade C	40%	65%	85%

**Table 10:** Results of BP level classification according to ESH/ESC guidelines.

Class	Accuracy	Sensitivity	Specificity	F1-Score	Actual Class Members
Grade 1 Hypertension	91.9%	75.8%	95.6%	77.9%	18.9%
Grade 2 Hypertension	97.7%	66.0%	99.3%	73.6%	4.9%
Grade 3 Hypertension	99.8%	25.6%	99.9%	28.6%	0.1%
High Normal	87.5%	73.3%	91.4%	71.4%	21.3%
Isolated Systolic Hypertension	97.9%	29.3%	98.9%	28.9%	1.4%
Normal	86.0%	72.8%	89.8%	70.1%	22.5%
Optimal	93.1%	87.5%	95.6%	88.6%	30.8%
Average	90.3%	76.9%	93.5%	77.0%	

## Data Availability

Publicly available datasets were analyzed in this study. This data can be found in [31]. The code developed and used in this work is available under request.

## References

[1] Fan Y., Xu P., Jin H., Ma J., Qin L. (2019). Vital Sign Measurement in Telemedicine Rehabilitation Based on Intelligent Wearable Medical Devices. IEEE Access.

[2] Pintavirooj C., Keatsamarn T., Treebupachatsakul T. (2021). Multi-Parameter Vital Sign Telemedicine System Using Web Socket for COVID-19 Pandemics. Healthcare.

[3] De Palma L., Attivissimo F., Di Nisio A., Lanzolla A.M.L., Ragolia M.A., Spadavecchia M. Development of a web-based system for interfacing a portable Bluetooth vital sign monitor. Proceedings of the 2022 IEEE International Symposium on Medical Measurements and Applications (MeMeA).

[4] Celler B.G., Sparks R.S. (2015). Home Telemonitoring of Vital Signs-Technical Challenges and Future Directions. IEEE J. Biomed. Health Inform..

[5] Scarpetta M., Spadavecchia M., Andria G., Ragolia M.A., Giaquinto N. Simultaneous Measurement of Heartbeat Intervals and Respiratory Signal using a Smartphone. Proceedings of the 2021 IEEE International Symposium on Medical Measurements and Applications (MeMeA).

[6] Khoshmanesh F., Thurgood P., Pirogova E., Nahavandi S., Baratchi S. (2021). Wearable sensors: At the frontier of personalised health monitoring, smart prosthetics and assistive technologies. Biosens. Bioelectron..

[7] Arpaia P., Moccaldi N., Prevete R., Sannino I., Tedesco A. (2020). A Wearable EEG Instrument for Real-Time Frontal Asymmetry Monitoring in Worker Stress Analysis. IEEE Trans. Instrum. Meas..

[8] D’Alessandro V.I., De Palma L., Attivissimo F., Di Nisio A., Lanzolla A.M.L. U-Net convolutional neural network for multisource heterogeneous iris segmentation. Proceedings of the 2023 IEEE International Symposium on Medical Measurements and Applications (MeMeA).

[9] Manickam P., Mariappan S.A., Murugesan S.M., Hansda S., Kaushik A., Shinde R., Thipperudraswamy S.P. (2022). Artificial Intelligence (AI) and Internet of Medical Things (IoMT) Assisted Biomedical Systems for Intelligent Healthcare. Biosensors.

[10] Cheng Y.-H., Lech M., Wilkinson R.H. (2023). Simultaneous Sleep Stage and Sleep Disorder Detection from Multimodal Sensors Using Deep Learning. Sensors.

[11] Castaneda D., Esparza A., Ghamari M., Soltanpur C., Nazeran H. (2018). A review on wearable photoplethysmography sensors and their potential future applications in health care. Int. J. Biosens. Bioelectron..

[12] Longmore S.K., Lui G.Y., Naik G., Breen P.P., Jalaludin B., Gargiulo G.D. (2019). A Comparison of Reflective Photoplethysmography for Detection of Heart Rate, Blood Oxygen Saturation, and Respiration Rate at Various Anatomical Locations. Sensors.

[13] Tamura T., Maeda Y., Sekine M., Yoshida M. (2014). Wearable Photoplethysmographic Sensors-Past and Present. Electronics.

[14] López-Silva S.M., Giannetti R., Dotor M.L., Silveira J.P., Golmayo D., Miguel-Tobal F., Bilbao A., Galindo M., Martín-Escudero P. (2022). Heuristic algorithm for photoplethysmographic heart rate tracking during maximal exercise test. J. Med. Biol. Eng..

[15] Qananwah Q., Dagamseh A., Alquran H., Ibrahim K.S., Alodat M.D., Hayden O. (2020). A comparative study of photoplethysmogram and piezoelectric plethysmogram signals. Phys. Eng. Sci. Med..

[16] De Palma L., Scarpetta M., Spadavecchia M. Characterization of Heart Rate Estimation Using Piezoelectric Plethysmography in Time- and Frequency-domain. Proceedings of the 2020 IEEE International Symposium on Medical Measurements and Applications (MeMeA).

[17] Block R.C., Yavarimanesh M., Natarajan K., Carek A., Mousavi A., Chandrasekhar A., Kim C.-S., Zhu J., Schifitto G., Mestha L.K. (2020). Conventional pulse transit times as markers of blood pressure changes in humans. Sci. Rep..

[18] Geddes L., Voelz M., Babbs C., Bourl J., Tacker W. (1981). Pulse transit time as an indicator of arterial blood pressure. Psychophysiology.

[19] Elgendi M., Fletcher R., Liang Y., Howard N., Lovell N.H., Abbott D., Lim K., Ward R. (2019). The use of photoplethysmography for assessing hypertension. NPJ Digit. Med..

[20] Tarvirdizadeh B., Golgouneh A., Tajdari F., Khodabakhshi E. (2020). A novel online method for identifying motion artifact and photoplethysmography signal reconstruction using artificial neural networks and adaptive neuro-fuzzy inference system. Neural Comput. Applic..

[21] Arabameri M., Nazari R.R., Abdolshahi A., Abdollahzadeh M., Mirzamohammadi S., Shariatifar N., Barba F.J., Khaneghah A.M. (2019). Oxidative stability of virgin olive oil: Evaluation and prediction with an adaptive neuro-fuzzy inference system (ANFIS). J. Sci. Food Agric..

[22] Slapničar G., Mlakar N., Luštrek M. (2019). Blood Pressure Estimation from Photoplethysmogram Using a Spectro-Temporal Deep Neural Network. Sensors.

[23] Harfiya L.N., Chang C.C., Li Y.H. (2021). Continuous Blood Pressure Estimation Using Exclusively Photopletysmography by LSTM-Based Signal-to-Signal Translation. Sensors.

[24] Chowdhury M.H., Shuzan M.N.I., Chowdhury M.E.H., Mahbub Z.B., Uddin M.M., Khandakar A., Reaz M.B.I. (2020). Estimating Blood Pressure from the Photoplethysmogram Signal and Demographic Features Using Machine Learning Techniques. Sensors.

[25] Tjahjadi H., Ramli K. (2020). Noninvasive Blood Pressure Classification Based on Photoplethysmography Using K-Nearest Neighbors Algorithm: A Feasibility Study. Information.

[26] Hasanzadeh N., Ahmadi M.M., Mohammadzade H. (2020). Blood Pressure Estimation Using Photoplethysmogram Signal and Its Morphological Features. IEEE Sens. J..

[27] Hsu Y.C., Li Y.H., Chang C.C., Harfiya L.N. (2020). Generalized Deep Neural Network Model for Cuffless Blood Pressure Estimation with Photoplethysmogram Signal Only. Sensors.

[28] Kurylyak Y., Lamonaca F., Grimaldi D. A Neural Network-based method for continuous blood pressure estimation from a PPG signal. Proceedings of the 2013 IEEE International Instrumentation and Measurement Technology Conference (I2MTC).

[29] Attivissimo F., De Palma L., Di Nisio A., Scarpetta M., Lanzolla A.M.L. (2023). Photoplethysmography Signal Wavelet Enhancement and Novel Features Selection for Non-Invasive Cuff-Less Blood Pressure Monitoring. Sensors.

[30] Kira K., Rendell L.A. (1992). The feature selection problem: Traditional methods and a new algorithm. Assoc. Adv. Artif. Intell..

[31] Kononenko I., Robnik-Šikonja M. (1997). Overcoming the myopia of inductive learning algorithms with RELIEFF. Appl. Intell..

[32] Roffo G. (2017). Ranking to learn and learning to rank: On the role of ranking in pattern recognition applications. arXiv.

[33] Ding C., Peng H. (2005). Minimum redundancy feature selection from microarray gene expression data. J. Bioinform. Comput. Biol..

[34] Moody B., Moody G., Villarroel M., Clifford G.D., Silva I. (2020). MIMIC-III Waveform Database (version 1.0). PhysioNet.

[35] Johnson A.E.W., Pollard T.J., Shen L., Lehman L.H., Feng M., Ghassemi M., Moody B., Szolovits P., Celi L.A., Mark R.G. (2016). MIMIC-III, a freely accessible critical care database. Sci. Data.

[36] Goldberger A.L., Amaral L.A., Glass L., Hausdorff J.M., Ivanov P.C., Mark R.G., Mietus J.E., Moody G.B., Peng C.K., Stanley H.E. (2000). PhysioBank, PhysioToolkit, and PhysioNet: Components of a new research resource for complex physiologic signals. Circulation.

[37] Kachuee M., Kiani M.M., Mohammadzade H., Shabany M. Cuff-less high-accuracy calibration-free blood pressure estimation using pulse transit time. Proceedings of the 2015 IEEE International Symposium on Circuits and Systems (ISCAS).

[38] Kachuee M., Kiani M.M., Mohammadzade H., Shabany M. (2017). Cuffless blood pressure estimation algorithms for continuous health-care monitoring. IEEE Trans. Biomed. Eng..

[39] Chakraborty A., Goswami D., Mukhopadhyay J., Chakrabarti S. (2021). Measurement of Arterial Blood Pressure Through Single-Site Acquisition of Photoplethysmograph Signal. in IEEE Trans. Instrum. Meas..

[40] Li Z., He W. (2021). A Continuous Blood Pressure Estimation Method Using Photoplethysmography by GRNN-Based Model. Sensors.

[41] Pandey R.K., Lin T.Y., Chao P.C.P. (2021). Design and implementation of a photoplethysmography acquisition system with an optimized artificial neural network for accurate blood pressure measurement. Microsyst. Technol..

[42] Guo J., Yang L., Bie R., Yu J., Gao Y., Shen Y., Kos A. (2019). An XGBoost-based physical fitness evaluation model using advanced feature selection and Bayesian hyper-parameter optimization for wearable running monitoring. Comput. Netw..

[43] Prabha A., Yadav J., Rani A., Singh V. (2022). Intelligent estimation of blood glucose level using wristband PPG signal and physiological parameters. Biomed. Signal Process. Control.

[44] Che X., Li M., Kang W., Lai F., Wang J. Continuous Blood Pressure Estimation from Two-Channel PPG Parameters by XGBoost. Proceedings of the 2019 IEEE International Conference on Robotics and Biomimetics (ROBIO).

[45] Shin H. (2022). XGBoost Regression of the Most Significant Photoplethysmogram Features for Assessing Vascular Aging. IEEE J. Biomed. Health Inform..

[46] Gao L., Ding Y. (2020). Disease prediction via Bayesian hyperparameter optimization and ensemble learning. BMC Res. Notes.

[47] Gregg M.E., Matyas T.A., James J.E. (2002). A new model of individual differences in hemodynamic profile and blood pressure reactivity. Psychophysiology.

[48] Sherwood A., Dolan C.A., Light K.C. (1990). Hemodynamics of blood pressure responses during active and passive coping. Psychophysiology.

[49] DeMers D., Wachs D. (2022). Physiology, mean arterial pressure. StatPearls.

[50] Kim J.Y., Cho B.H., Im S.M., Jeon M.J., Kim I.Y., Kim S.I. Comparative study on artificial neural network with multiple regressions for continuous estimation of blood pressure. Proceedings of the 2005 IEEE Engineering in Medicine and Biology 27th Annual Conference.

[51] Cattivelli F.S., Garudadri H. Noninvasive cuffless estimation of blood pressure from pulse arrival time and heart rate with adaptive calibration. Proceedings of the 2009 Sixth International Workshop on Wearable and Implantable Body Sensor Networks.

[52] Zhang Y., Feng Z. A SVM method for continuous blood pressure estimation from a PPG signal. Proceedings of the 9th International Conference on Machine Learning and Computing.

[53] Zadi A.S., Alex R., Zhang R., Watenpaugh D.E., Behbehani K. (2018). Arterial blood pressure feature estimation using photoplethysmography. Comput. Biol. Med..

[54] Wang L., Zhou W., Xing Y., Zhou X. (2018). A Novel Neural Network Model for Blood Pressure Estimation Using Photoplethesmography without Electrocardiogram. J. Healthc. Eng..

[55] Fleischhauer V., Feldheiser A., Zaunseder S. (2022). Beat-to-Beat Blood Pressure Estimation by Photoplethysmography and Its Interpretation. Sensors.

[56] Liu M., Po L.-M., Fu H. (2017). Cuffless blood pressure estimation based on photoplethysmography signal and its second derivative. Int. J. Comput. Theory Eng..

[57] Zhang G., Shin S., Jung J., Li M., Kim Y.T. Machine learning Algorithm for Non-invasive Blood Pressure Estimation Using PPG Signals. Proceedings of the 2022 IEEE Fifth International Conference on Artificial Intelligence and Knowledge Engineering (AIKE).

[58] Stergiou G.S., Alpert B., Mieke S., Asmar R., Atkins N., Eckert S., Frick G., Friedman B. (2018). A universal standard for the validation of blood pressure measuring devices: Association for the Advancement of Medical Instrumentation/European Society of Hypertension/International Organization for Standardization (AAMI/ESH/ISO) Collaboration Statement. Hypertension.

[59] (2003). Association for the Advancement of Medical Instrumentation, American National Standard. Manual, Electronic or Automated Sphygmomanometers.

[60] O’brien E., Waeber B., Parati G., Staessen J., Myers M.G. (2001). Blood pressure measuring devices: Recommendations of the European Society of Hypertension. BMJ.

[61] Rong M., Li K. (2021). A multi-type features fusion neural network for blood pressure prediction based on photoplethysmography. Biomed. Signal Process. Control.

[62] Li Y.H., Harfiya L.N., Chang C.C. (2021). Featureless Blood Pressure Estimation Based on Photoplethysmography Signal Using CNN and BiLSTM for IoT Devices. Hindawi Wirel. Commun. Mob. Comput..

[63] Mousavi S.S., Firouzmand M., Charmi M., Hemmati M., Moghadam M., Ghorbani Y. (2019). Blood pressure estimation from appropriate and inappropriate PPG signals using A whole-based method. Biomed. Signal Process. Control.

[64] Mancia G., Fagard R., Narkiewicz K., Redon J., Zanchetti A., Böhm M., Christiaens T., Cifkova R., De Backer G., Dominiczak A. (2013). 2013 ESH/ESC Guidelines for the management of arterial hypertension: The Task Force for the management of arterial hypertension of the European Society of Hypertension (ESH) and of the European Society of Cardiology (ESC). Eur. Heart J..

[65] Altman D., Bland J. (1983). Measurement in Medicine: The Analysis of Method Comparison Studies. Statistician.

[66] Dogan N. (2018). Bland-Altman analysis: A paradigm to understand correlation and agreement. Turk. J. Emerg. Med..

[67] Giavarina D. (2015). Understanding Bland Altman analysis. Biochem. Medica.

